# The good, the bad, and the environment: developing an area-based measure of access to health-promoting and health-constraining environments in New Zealand

**DOI:** 10.1186/s12942-021-00269-x

**Published:** 2021-04-06

**Authors:** Lukas Marek, Matthew Hobbs, Jesse Wiki, Simon Kingham, Malcolm Campbell

**Affiliations:** 1 GeoHealth Laboratory, Geospatial Research Institute, University of Canterbury, Christchurch, New Zealand; 2 School of Health Sciences, University of Canterbury, Christchurch Canterbury, New Zealand; 3 School of Earth and Environment, University of Canterbury, Christchurch, Canterbury, New Zealand

**Keywords:** Accessibility, Built environment, Food environment, GIS, Health, Healthy cities, Natural environment

## Abstract

**Background:**

Accounting for the co-occurrence of multiple environmental influences is a more accurate reflection of population exposure than considering isolated influences, aiding in understanding the complex interactions between environments, behaviour and health. This study examines how environmental ‘goods’ such as green spaces and environmental ‘bads’ such as alcohol outlets co-occur to develop a nationwide area-level healthy location index (HLI) for New Zealand.

**Methods:**

Nationwide data were collected, processed, and geocoded on a comprehensive range of environmental exposures. Health-constraining ‘bads’ were represented by: (i) fast-food outlets, (ii) takeaway outlets, (iii) dairy outlets and convenience stores, (iv) alcohol outlets, (v) and gaming venues. Health-promoting ‘goods’ were represented by: (i) green spaces, (ii) blue spaces, (iii) physical activity facilities, (iv) fruit and vegetable outlets, and (v) supermarkets. The HLI was developed based on ranked access to environmental domains. The HLI was then used to investigate socio-spatial patterning by area-level deprivation and rural/urban classification.

**Results:**

Results showed environmental ‘goods’ and ‘bads’ co-occurred together and were patterned by area-level deprivation. The novel HLI shows that the most deprived areas of New Zealand often have the most environmental ‘bads’ and less access to environmental ‘goods’.

**Conclusions:**

The index, that is now publicly available, is able to capture both inter-regional and local variations in accessibility to health-promoting and health-constraining environments and their combination. Results in this study further reinforce the need to embrace the multidimensional nature of neighbourhood and place not only when designing health-promoting places, but also when studying the effect of existing built environments on population health.

**Supplementary Information:**

The online version contains supplementary material available at 10.1186/s12942-021-00269-x.

## Introduction

Exposure to features of the built and natural environment within a local neighbourhood is known to facilitate and constrain individual choices regarding health-related behaviours and affect subsequent health outcomes [1, 2]. A proliferation of particular environmental features in one area may influence behavioural choices via easy and convenient access as well as via other means such as influencing social norms [3–7]. Moreover, the environment has the potential to make decisions subconscious or automatic [8–10], a response termed passive over-consumption [8, 11, 12]. Importantly, this relates to features that are considered health-constraining as well as those considered health-promoting.

Based on current literature, health-constraining ‘bads’ are considered as stores predominately selling energy-dense and nutrient-poor foods including fast food outlets [13–15], convenience stores—otherwise known as a ‘dairy’ in New Zealand [13, 14, 16–21], and takeaway shops [22]. These ‘bads’ are not restricted to food outlets alone however, as alcohol outlets and gambling/gaming venues [7, 23–26] are also considered to be health-constraining based on the broader social harms associated with these outlets. In contrast, health-promoting ‘goods’ are considered as stores that sell fresh produce including fruit/vegetable stores, green grocers and grocery stores as well as supermarkets [22, 27]. Health-promoting ‘goods’ also consider physical activity facilities [28–30] as well as natural environments used for physical activity and recreation such as green space [31–36] and blue space [37–40]. Other notable features of the environment include land use mix, street typology and walkability; these affect how a place is designed and navigated and can be influential for health [30].

While the environment influences many human behaviours, research suggests that such the built environment is not evolving equitably across social strata with those in more deprived areas having poorer local environments to reside and work within [22, 28]. A disproportionate number of unhealthy food outlets are located in socioeconomically deprived areas and areas with high ethnic minority populations [41–46]. Other research has shown higher levels of environmental concerns such as graffiti and litter in more deprived areas [47]. Furthermore, even if an individual does have access to a favourable physical activity environment with green spaces and physical activity facilities available, it may be the more affluent that benefit due to economic accessibility [48]. Indeed, the ability for an individual adopt a healthier diet or be physically active may not be attributable to psychosocial factors and is perhaps more heavily influenced by the socioeconomic position of the household and the environment which surrounds them [49]. It is also plausible that multiple environmental exposures act simultaneously and interdependently to influence geographical differences in health behaviours and outcomes.

A plethora of evidence, with a diverse range of different methods, has investigated links between environmental features, social outcomes, and health outcomes [16, 30]. With increasing attention paid to the inclusion of multiple environmental features, recent studies have developed small area indices or used relative measures such as ratios and proportions [12, 27, 50]. There is no clear consensus on the most appropriate measure, yet there is evidence to suggest that environmental features such as fast-food and alcohol outlets co-occur or cluster within neighbourhoods [26, 51, 52]. Therefore, it is worthy to not treat each environmental exposure in isolation [52]. Such factors may also represent a complex system where dependencies, interactions and relationships give rise to larger collective behaviours or exhibit non-stationarity and non-linearity [53]. While the evidence base around multiple environmental exposures is developing with no recognised gold standard [16], there is a need to add to this in order to accurately demonstrate how the wider environment influences human behaviour and health. We therefore respond to calls from other researchers who request a continued search for better ways of representing population exposure to the environment [50, 54].

Currently, in addition to considering environmental exposures in isolation, evidence is restricted by several limitations. First, the majority of studies are from North America where the structure of the built environment and zoning regulations differ from New Zealand and many other countries. Second, nationwide data is often difficult to acquire and therefore seldom considered. Third, providing evidence for New Zealand is particularly important as it is a country with increasing spatial inequalities in health [55, 56]. Finally, there is further need to assess the co-occurrence of a comprehensive range of environmental exposures that are both health-constraining ‘bads’ and health-promoting ‘goods’. This study first aims to examine how health-constraining ‘bads’ (fast-food outlets, takeaway outlets, dairy outlets and convenience stores, alcohol outlets, and gaming venues) as well as health-promoting ‘goods’ (green spaces, blue spaces, physical activity facilities, supermarkets, and fruit and vegetable outlets) co-occur in the same geographical areas and outline the development of the combined index [the healthy location index (HLI)]. Second, it examines socio-spatial patterning of the health-promoting ‘goods’ and health-constraining ‘bads’ by area-level deprivation and urbanicity of the area.

## Methods

### Environmental data

We collected, cleaned, processed, and validated data on ten environmental ‘goods’ and ‘bads’ from several sources including Territorial Authorities, Ministry for Primary Industries (MPI), Ministry for the Environment (MfE), Land Information New Zealand (LINZ), Alcohol Regulatory and Licensing Authority, Zenbu, and Department of Internal Affairs for the period of 2015–2018. We follow the approach presented by Wilkins et al. (2017) and followed the Geo-FERN reporting framework (shown in Additional file 1) [57]. Table 1 shows the summary of environmental features included in the study and key methodological details.

**Table 1:** A summary of the environmental data used, data source, and time frame

Environment data	Extracting features	Defining constructs	Geocoding methods	Access metrics/other detail
Supermarkets Ministry for Primary Industries (2017) Territorial Authorities (2013–2015)	Extracted based on propriety classifications provided by MPI/TA and the business name of multinational franchises		Geocoded to address level 99% match rate using Google API	Full street address provided
Fast food Ministry for Primary Industries (2017) Territorial Authorities (2013–2015)	Extracted based on propriety classifications provided by MPI/TA and the business name of multinational franchises	Food outlets: businesses that manufacture, prepare, or sell food or food products are required to be registered through the Ministry for Primary Industries Food Control Programme and/or Territorial Authorities Health licenses	Geocoded to address level 99% match rate using Google API	Full street address provided
Takeaway Ministry for Primary Industries (2017) Territorial Authorities (2013–2015)	Extracted based on propriety classifications provided by MPI/TA and the business name of national businesses providing takeaway food options		Geocoded to address level 99% match rate using Google API	Full street address provided
Dairy/convenience Ministry for Primary Industries (2017) Territorial Authorities (2013–2015)	Extracted based on propriety classifications provided by MPI/TA and the business name of retailers selling pre-packaged convenience foods (includes dairy, convenience, superette, service stations, small grocers)		Geocoded to address level 99% match rate using Google API	Full street address provided
Fruit and vegetable Ministry for Primary Industries (2017) Territorial Authorities (2013–2015)	Extracted based on propriety classifications provided by MPI/TA and business name (includes fruit/vegetable stores, produce retailers, green grocers)		Geocoded to address level 98% match rate using Google API	Full street address provided
Physical activity facilities Territorial Authorities (2013–2015) Zenbu Directory (2006–2017)	Extracted based on propriety classifications provided by TA/Zenbu (includes gym, fitness centre, sports hall, tennis, rugby, swimming, soccer, bowls, golf, hockey, cricket, martial arts, rowing)	Activity facilities: facilities where physical activity is the main purpose:	Geocoded to address level 98% match rate using Google API	Location of activity facilities only. Does not contain sports parks due to their similarity with green space Not systematically registered through government bodies
Alcohol outlets Alcohol Regulatory and Licensing Authority (ARLA) (2015–2018) Licence register	Extracted based on proprietary classifications provided by ARLA	Alcohol outlets: in NZ the sale of alcohol to the public requires the seller to have a licence	Geocoded to address level 92% match rate achieved in ArcGIS geocoding which were correct to the CAU level	x, y coordinate provided Alcohol outlet address data had a high prevalence of missing data which required manual processing
Gaming facilities 2018 Department of Internal Affairs register	Extracted based on proprietary classification in register for all licenced gaming venues	Defined consistent with the Department of Internal Affairs register	Data were geocoded using Google Maps Geocoding API through R and ggmap package (98% match rate)	Full street address provided
Green spaces Extracted in March 2019 from Land Cover Database (LCDB) V5.0	Extracted based on proprietary classifications in LCDB	Indigenous Forest, Mangrove, Exotic Forest, Gorse and/or Broom, Deciduous Hardwoods, Manuka and/or Kanuka,Urban Parkland/Open Space, Broadleaved Indigenous Hardwoods, Matagouri or Grey Scrub, Sub Alpine Shrubland, Fernland, Herbaceous Saline Vegetation, Mixed Exotic Shrubland, Alpine Grass/Herbfield, Herbaceous Freshwater Vegetation, Flaxland	Official layer extracted from the New Zealand Land Cover Database Version 5.0	N/A
Blue spaces Extracted in March 2019 from Land Cover Database (LCBD) V5.0 and WaterAreas Topo50	Extracted based on proprietary classifications in LCDB	Lake or Pond, River, Estuarine Open Water, Lake, Lagoon, Reservoir, Pond, and Canal, including coastline	Official layer extracted from the New Zealand Land Cover Database Version 5.0	N/A

#### Food outlets and physical activity facilities

In New Zealand, all businesses that manufacture, prepare, or sell food or food products have to register through the Ministry for Primary Industries (MPI) Food Control Programme (FCP)—previously the Food Safety Programme (FSP). National data on business registrations were collected from MPI sources in August of 2017 and include the food service sector and food retailers. Sectors not included relate primarily to the nature of the business such as manufacturers and processors. Records with a valid registration date were kept from the FSP, then combined with data collected from the FCP to create the final MPI dataset.

Territorial Authorities (TAs) are responsible for uploading data to the FCP from March 2016 however, with many TAs yet to upload their data. To ensure a comprehensive dataset, 66 TAs (the Chatham Islands were excluded due to the remote nature of their geography) were contacted individually and. data on health licenses from 2013 to 2015 were requested. Where given, pre-defined proprietary classifications of business were used to categorise each business (89.6%). If the business category was not given then the business name was used for categorisation, with ambiguous names investigated in further detail or excluded if a category could not be clearly defined.

Data on supermarkets (n = 571), fast food outlets (n = 754), takeaway outlets (n = 2,428), dairy/convenience outlets (n = 2,130), and fruit and vegetable stores (n = 223) were collected from two data sources, TAs and MPI. Data from TAs has been used in the majority of research that investigates environmental exposures in New Zealand [58, 59]. Yet, many TAs noted that they had either uploaded data to MPI’s FCP or that their data was incomplete given that they do not register many of the larger multinational chains, which is the responsibility of MPI. Therefore a combination of sources was used to ensure a comprehensive dataset.

Data on physical activity facilities was collected from TAs and Zenbu business directory in August of 2017. Zenbu is a crowd-sourced business directory that contains records from 2006 onward. It was used to supplement data on activity facilities, where only a small amount of data was available from government bodies. The activity facilities category consisted of n = 1189 records in total (n = 334 from TAs, n = 855 from Zenbu).

In total n = 38,875 records on food outlets and physical activity facilities were received; n = 21,392 from Territorial Authorities (TAs), n = 16,610 records from the Ministry for Primary Industries, and n = 855 records from Zenbu. If duplicate entries for one premise were given only the latest registration was kept, however, duplicate business names for current registrations at different locations were kept as unique records. After removing closed premises (n = 2823), duplicated premises (n = 9406), and premises with insufficient spatial information (n = 2839) the resulting dataset consisted of n = 23,789 records.

Categories which were included in this study are detailed in Table 1, consisting of n = 6724 total records. Data were validated by type and consistency for all sources. Classification of the categories included were fairly consistent across government entities and cross-reference validation was used for verification of data sourced from more than one provider. In case of individual source data, we validated 100 random records using other resources such as online maps. Records from these study categories were then geocoded using Google Maps Application Programming Interface (API), at the address level of precision. The geographic coordinates for any un-matched records in this category were manually investigated (n = 43). Unmatched records were more commonly observed in rural areas compared to urban areas as the full street address including street number was often not provided and address was unable to be retrieved manually. One hundred random records were tested to assess the accuracy of geocoding (see Table 1 for details).

#### Alcohol outlets

Alcohol outlets were sourced from the Alcohol Regulatory and Licensing Authority (ARLA) for the period 2015–2018 from the current and active licence register. All alcohol outlets (n = 19,035) were extracted from the database based on the proprietary classifications provided by ARLA. The sale of alcohol to the public in NZ requires the seller to have a licence. Data were removed if duplicates (n = 3657) or if conveyance services (n = 203) such as airlines who were often registered at the airport rather than the actual location of sale. Highways were then checked manually (n = 165) as address records often stated the business name and state highway 1 for instance. Subsequently, 13 records were removed as addresses could not be identified. This resulted in a final sample of 13,990 alcohol outlets to geocode within ArcGIS. Within the output, 13,695 were matched and 295 produced tied results. We then tested a random 100 records to investigate if they were geocoded to the correct census area unit (CAU) and 92% were found to be correct.

#### Gaming venues

Data on the location of gaming venues (n = 1081) were obtained from the 2018 Department of Internal Affairs register and were extracted based on proprietary classification in the register for all licenced gaming venues. Constructs were defined as consistent with the Department of Internal Affairs register and included licensed venues operating gaming machines Data were geocoded using Google Maps Geocoding API through Rstudio using the ggmap package with one record not being able to geocode. As in with previous data, we have tested 100 randomly selected record in order to estimate geocoding accuracy (98%).

#### Green spaces and blue spaces

Data on green and blue spaces were obtained from the Land Cover Database (LCDB) V5.0 and WaterAreas Topo50, and were accurate as of March 2019. While green spaces were extracted based on the proprietary classifications in LCDB (Table 1), combination of LCDB and WaterAreas 50 was used to extract more comprehensive blue spaces including coastlines. After extraction, both datasets were transformed to 50 × 50 m raster grid. Spatial and database operations were undertaken using QGIS 3.12 [60].

#### Defining access to environmental features

For the analysis of accessibility, we used meshblock administrative units that are the smallest geographic units for which statistical data is reported by Stats NZ with ideal size range of 30–60 dwellings (around 60–120 residents) [61]. There were 52,923 meshblocks (oceanic meshblocks excluded) included in the analysis [median area = 0.05 km^2^, mean area = 5.06 (4.79–5.34) km^2^]. For all features other than blue and green spaces distance from the 2018 population-weighted centroid of the 2018 meshblock were calculated via road network [62] using ArcGIS Pro v2.4 [63]. For blue and green spaces, median proximity from any place in the meshblock to each blue and green space (Euclidean distance) based on the 50 × 50 m grid was calculated for each meshblock instead of the closest facility. This approach was utilised due to (1) green and blue spaces being an area phenomenon; (2) there may not be an exact address or place of entrance; (3) even proximity and visibility of green and blue spaces may have health-promoting effects.

Data were then classified as health-constraining ‘bads’ (fast-food outlets, takeaway outlets, dairy outlets and convenience stores, alcohol outlets, and gaming venues) and health-promoting ‘goods’ (green spaces, blue spaces, physical activity facilities, supermarkets, and fruit and vegetable outlets). There are a plethora of existing complex methods used for the construction of indices in socioeconomic geography; some of them utilising data transformation and weighting schemes [64], regression models [65], multivariate statistics [66] or multi-criteria decision analysis [67]. However, we opted for more straightforward method of creating typology based on the domain rankings that, to some extent, follows several papers which use this approach [64, 68]. This approach provided a non-parametrical basis for the analysis as individual domains differed in their distributions. At the same time, it gave easy to understand metric, allowing immediate comparison between domains. We have not used any weights of domains due to unequivocal theoretical reasoning for them.

To construct indices of environmental ‘goods’ and ‘bads’, each meshblock was ranked based on its access to the closest individual environmental features in all domains except green- and blue spaces (values from 1 to 52,923, one being the closest to the feature). The proximity measure was used for ranking of green and blue spaces. Then, ranks for health-promoting ‘goods’ (green spaces, blue spaces, physical activity facilities, supermarkets and fruit and vegetable outlets) and health-constraining ‘bads’ (fast-food outlets, takeaway outlets, dairy outlets and convenience stores, alcohol outlets, and gaming venues) were summed. These scores were ranked again to get information about combined access to environmental ‘goods’ and ‘bads’. As the final step, deciles were assigned to ranks. The resulting index is between 1 and 10. Decile 1 was defined as the best accessibility while Decile 10 was defined as the worst accessibility. For ‘goods’ this meant that the best accessibility was healthy, for instance with greater access to ‘goods’ such as green spaces. For ‘bads’, greater accessibility was a bad thing as this means greater accessibility to environmental factors such as alcohol outlets.

#### Developing an area-based measure of access to health-promoting and health-constraining environments in New Zealand: a healthy location index

**Fig. 1:**
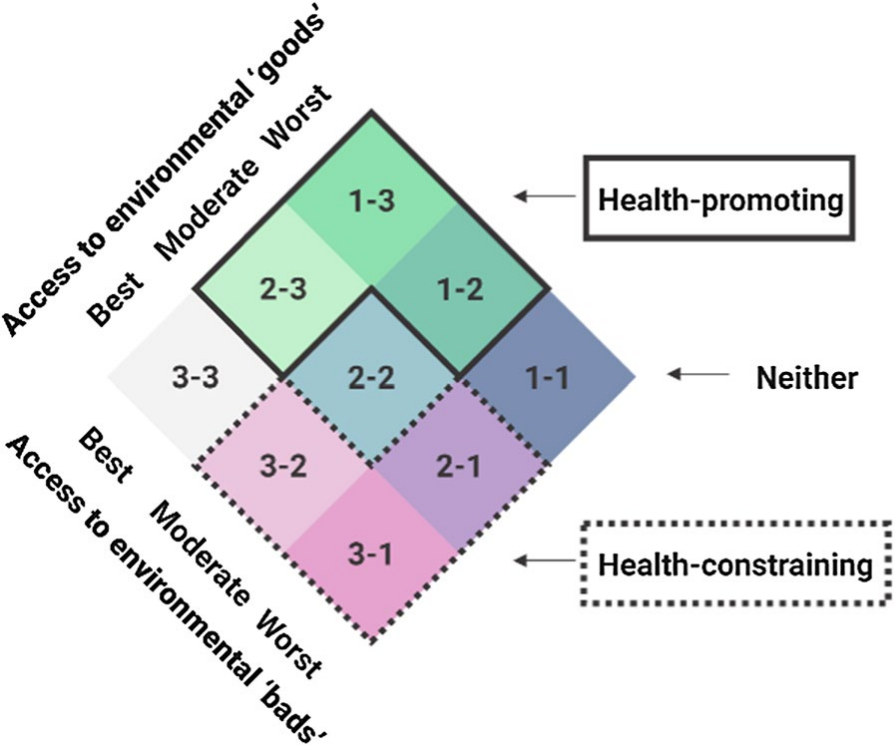
Final combination of access to health-promoting (‘goods’) and health-constraining (‘bads’) environments

To combine the environmental data in this study we first split environmental ‘goods’ and ‘bads’ deciles into three categories; category one was the best accessibility (deciles 1–3), category two was defined as mid accessibility (deciles 4–7), and category three was defined as the worst accessibility (deciles 8–10) of health-promoting and health-constraining environments. For environmental ‘goods’ category one is the most health-promoting environment while category three is the least health-promoting environment. For environmental ‘bads’ category one was the most health-constraining while category three was the least health-constraining. Data were then combined into nine possible combinations of environmental ‘goods’ and ‘bads’ to develop a healthy location index for the whole of New Zealand at the meshblock scale (Fig. 1).

#### Additional data

We utilised additional descriptive and demographic data provided by Statistics New Zealand to provide a comprehensive picture of areas and population characteristics in regard to the index. Firstly, we used the area-level deprivation defined as the NZdep2018 index of deprivation [66]. NZDep2018 reflects eight dimensions of material and social deprivation which reflect a lack of income, employment, communication, support, qualifications, owned home, living space, and dry living conditions. We used the ordinal scale which ranged from 1 to 10 (deciles), where 1 represented the areas with the least deprived scores and 10 the areas with the most deprived scores. In graphs, we used quintiles due to better visual clarity of the outputs. Further, we used population counts in meshblock based on Census 2018 to estimate population sums and Urban Rural Indicator (official urban–rural classification) to distinguish between categories of urban and rural space [69] such as major urban areas (population of 100,000 or more), large urban areas (30,000–99,999), medium urban areas (10,000–29,999) and small urban areas (1000–9999), rural settlements (200–999) and other rural areas.

**Fig. 2:**
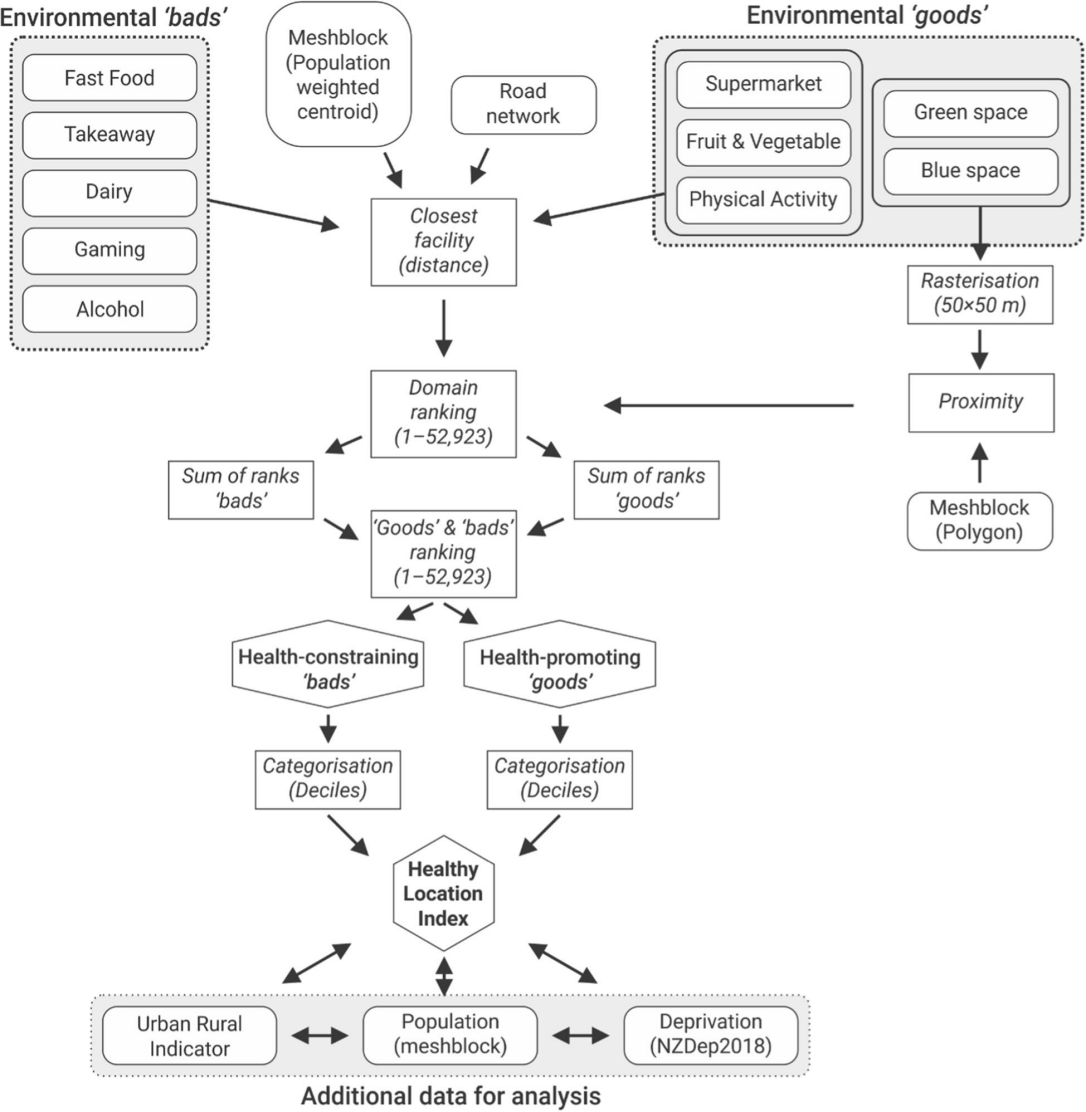
Data and methods used in the development of the healthy location index

The following conceptual diagram (Fig. 2) summarises data and methods used in the process of creation of HLI including additional datasets used in the further analyses of the index.

## Results

### Descriptive statistics

Table 2 shows the median distance to health-constraining environmental ‘bads’ including fast-food (2.28 km), takeaway (1.30 km), dairy and convenience (1.18 km), alcohol (0.68 km) and gaming venues (1.65 km) across New Zealand. Social gradients were displayed across all outlets with proximity to environmental ‘bads’ generally decreasing as area-level deprivation increased. An exception was often noted for the most deprived decile with a small increase in distance. For example, the least deprived (D1) and second least deprived decile (D2) were 1.94 km and 1.79 km from takeaway outlets, respectively. In contrast, the most deprived (D10) and second most deprived decile (D9) were a median of 0.97 km and 0.84 km from takeaway outlets. Table 2 also shows the median distance to health-promoting environmental ‘goods’ including physical activity facilities (1.60 km), fruit and vegetable outlets (4.59 km), green spaces (0.14 km), blue spaces (0.64 km), and supermarkets (1.81 km). With the exception of an increase in the most deprived decile, distance to physical activity facilities, fruit and vegetable outlets, and supermarkets were clearly patterned by area-level deprivation with reduced proximity in the least deprived areas. While this pattern was mirrored in supermarkets and fruit and vegetable outlets, the differences noted for blue and green spaces were less pronounced than in the other ‘goods’. More details on the distribution of distances in all domains is provided in Additional file 1: Figure S1.

**Table 2:** Median distance to environmental ‘goods’ and ‘bads’ in New Zealand (in km)

NZDep 2018 deprivation decile	Environmental ‘bads’					Environmental ‘goods’				
	Fast-food	Takeaway	Dairy	Alcohol	Gaming	Physical activity facilities	Fruit and vegetable outlets	Green spaces	Blue spaces	Supermarkets
Overall	2.28	1.30	1.18	0.68	1.65	1.60	4.59	0.14	0.64	1.81
D1 (least deprived)	3.06	1.94	1.88	0.95	2.53	2.07	5.53	0.11	0.56	2.32
D2	2.85	1.79	1.68	0.89	2.34	1.94	5.24	0.14	0.57	2.26
D3	2.86	1.74	1.59	0.84	2.13	1.91	5.45	0.14	0.62	2.21
D4	2.60	1.55	1.47	0.78	1.95	1.78	5.02	0.14	0.65	2.04
D5	2.42	1.38	1.24	0.73	1.72	1.64	5.11	0.14	0.65	1.88
D6	2.43	1.34	1.27	0.69	1.67	1.71	5.97	0.15	0.67	1.87
D7	1.97	1.10	0.97	0.61	1.42	1.46	4.42	0.15	0.66	1.66
D8	1.70	0.92	0.82	0.53	1.19	1.28	3.81	0.15	0.64	1.44
D9	1.54	0.84	0.72	0.50	1.10	1.20	3.17	0.15	0.66	1.30
D10 (most deprived)	1.94	0.97	0.87	0.58	1.33	1.47	3.34	0.11	0.67	1.44
*Significance	< 0.001	< 0.001	< 0.001	< 0.001	< 0.001	< 0.001	< 0.001	< 0.001	< 0.001	< 0.001

^*^Significance denotes results of test for group differences

### Co-occurrence of health-promoting environmental ‘goods’ and health-constraining ‘bads’

**Fig. 3:**
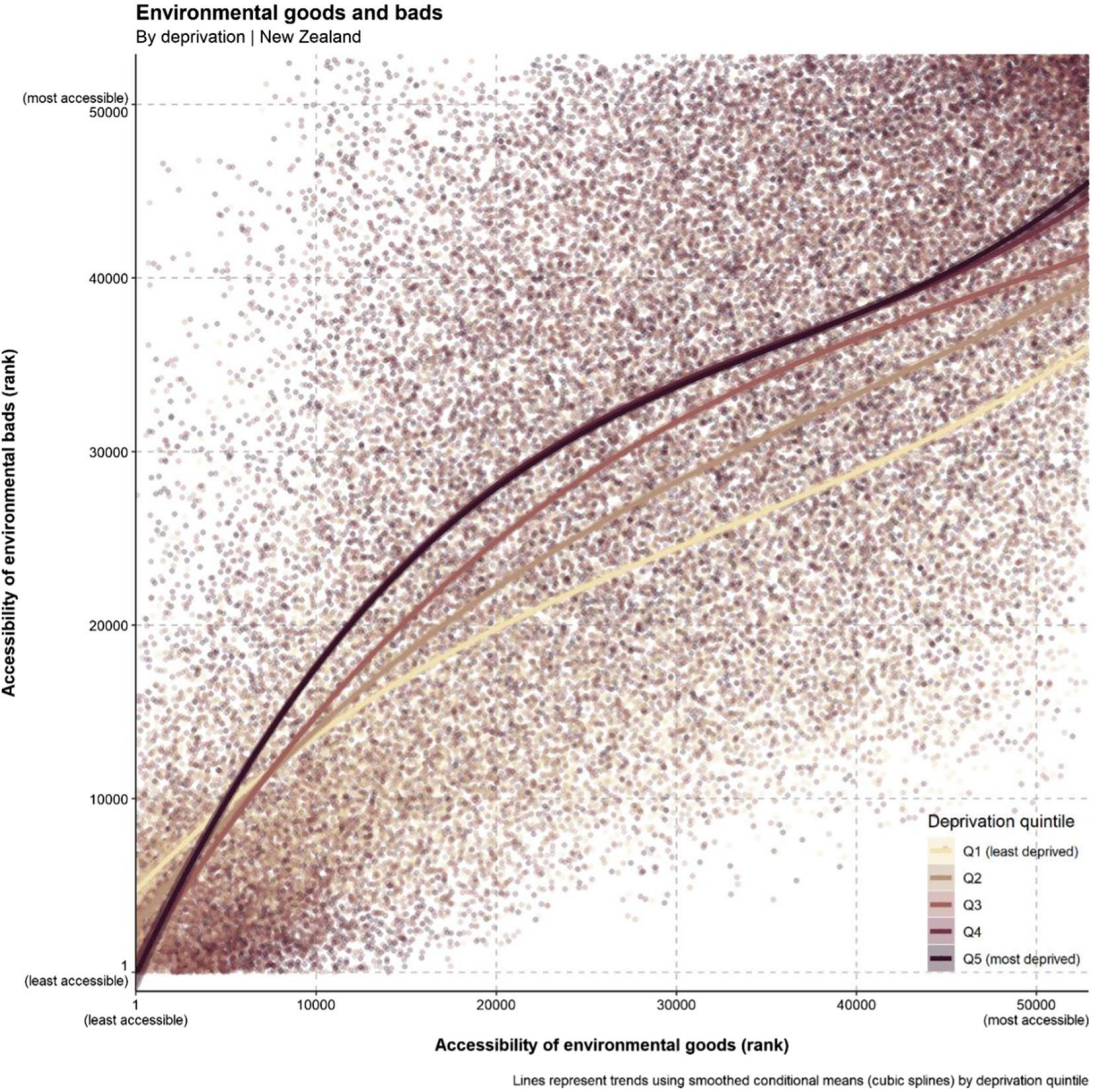
The correlation between accessibility to environmental ‘goods’ (rank) and environmental ‘bads’ (rank) by deprivation quintile (Quintile 5 (Q5) most deprived)

Figure 3 shows the overall association between accessibility to ranked environmental ‘goods’ and environmental ‘bads’ by deprivation quintile. The accessibility of environmental determinants increases alongside deprivation. As accessibility increases overall, the pattern of social stratification represented by area-level deprivation becomes more prominent. While the outliers in the bottom of the graph (below smoothed conditional means lines) are mostly less deprived meshblocks that have good accessibility to environmental ‘goods’ without improving access to environmental ‘bads’, the meshblocks in the upper part of the graph show an inverse pattern—more deprived meshblocks with good access to ‘bads’ and limited access to ‘goods’.

**Fig. 4:**
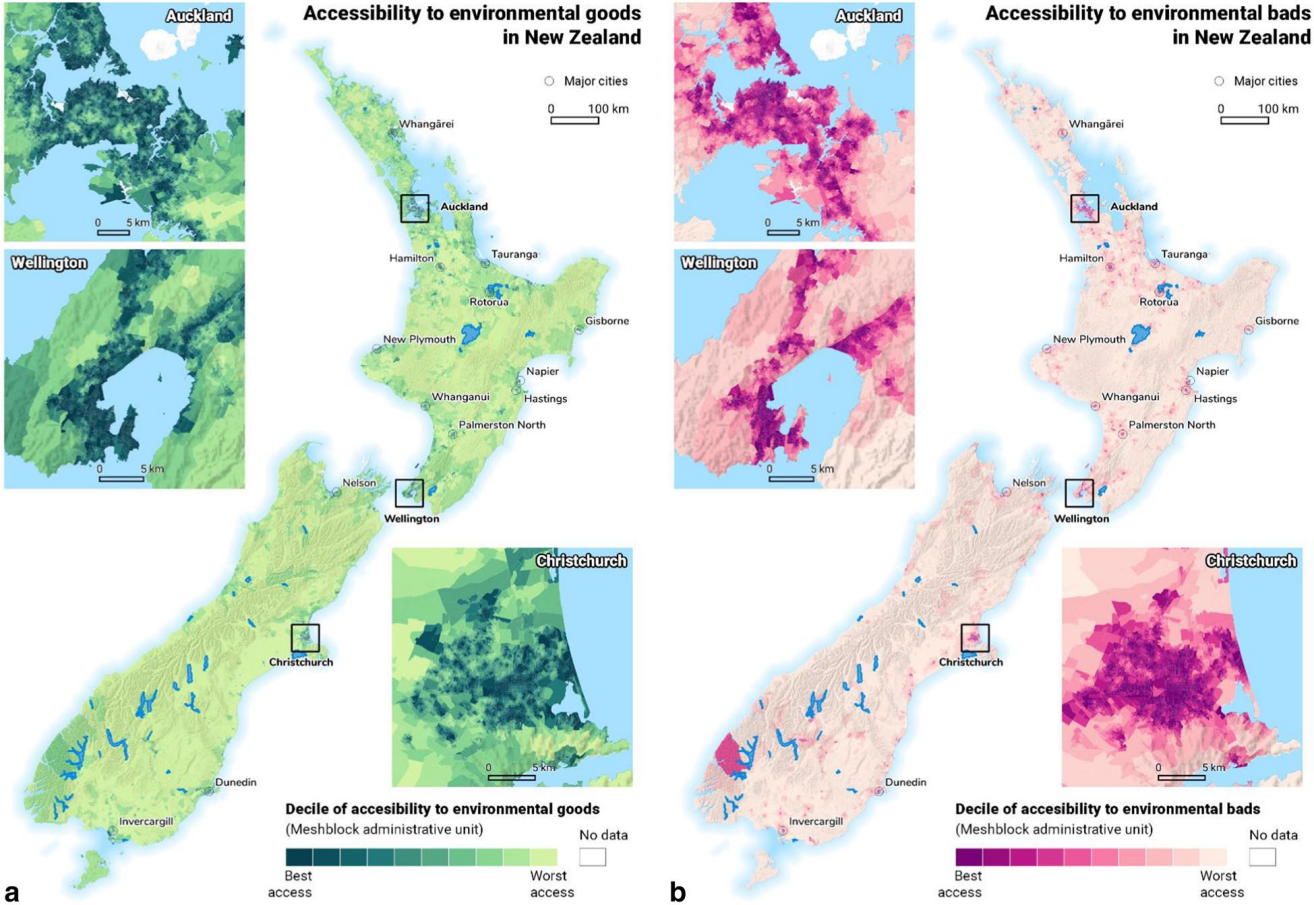
The spatial patterning of environmental ‘goods’ (**a**) and ‘bads’ (**b**) in New Zealand and urban areas of Auckland, Christchurch, and Wellington

While we constructed our index for the whole of New Zealand, Fig. 4a shows the co-occurrence of health-promoting environmental ‘goods’ in the three major cities of Wellington, Christchurch, and Auckland. Decile 1 shows the best accessibility to environmental features while decile 10 represents limited access. In this case, decile 1 is desirable as this represents the best access to environmental ‘goods’ such as green spaces and physical activity facilities. In contrast, decile 10 is undesirable. Figure 4b shows the co-occurrence of health-constraining environmental ‘bads’ in Wellington, Christchurch, and Auckland. Notably, decile 1 now shows the most ‘bads’ such as alcohol outlets and gaming venues and is undesirable while decile 10 has the least ‘bads’ and is more desirable. As shown in Fig. 4b, throughout New Zealand, high accessibility of both environmental ‘goods’ and ‘bads’ is evident in urban areas. Within such areas, the most central parts of the city and areas along the coastline demonstrate high access to environmental ‘goods’. The most central parts of the city also show particularly high access to environmental ‘bads’. In contrast, rural areas demonstrate poor or limited accessibility of both environmental ‘goods’ and ‘bads’. This spatial pattern is likely reflective of population density, centralised business districts that offer a wide range of facilities, and urban areas which also experience high exposure to blue spaces by being located in close proximity to the coastline.

### Development of the healthy location index (HLI)

**Fig. 5:**
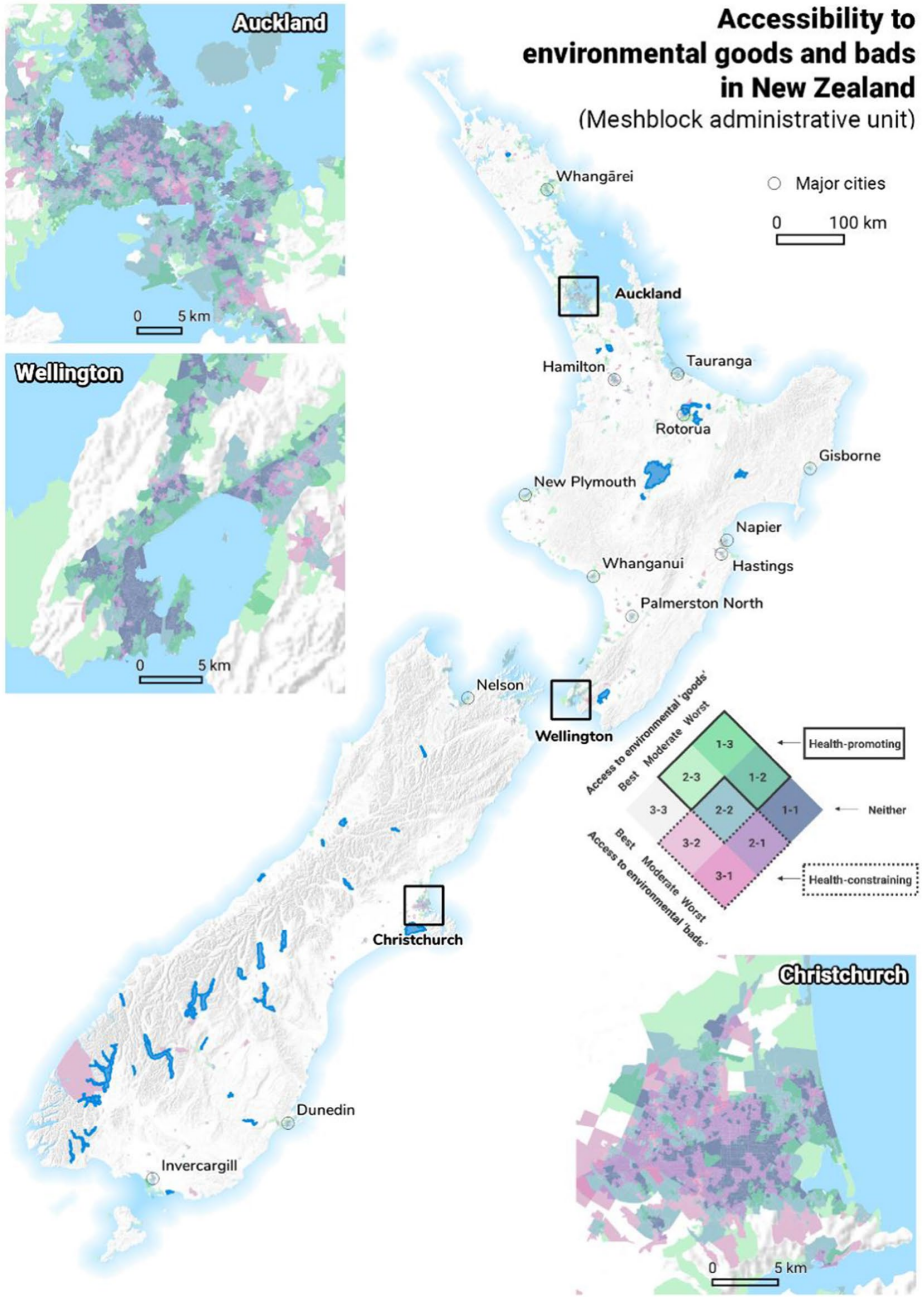
The spatial patterning of healthy location index (HLI) based on access to environmental ‘goods’ and ‘bads’ in New Zealand and urban areas of Auckland, Christchurch, and Wellington

Figure 5 shows the healthy location index (HLI) with nine possible categories that the HLI could be based on. For each category, 1 denotes the best access while 3 denotes the worst access. Therefore, for category 1–3 this denotes best access to environment goods and worst access to environmental bads. This category titled in dark blue [1–3] represents the most desirable or healthiest category of the index with the best access to environmental ‘goods’ and limited access to environmental ‘bads’. In contrast, the category in pink (3–1) represents the most undesirable or unhealthy category of the index with the best access to environmental ‘bads’ but worst access to environmental ‘goods’. Figure 5 also shows the co-occurrence of environmental ‘goods’ and ‘bads’ in the healthy location index for Wellington, Christchurch, and Auckland spatially.

**Fig. 6:**
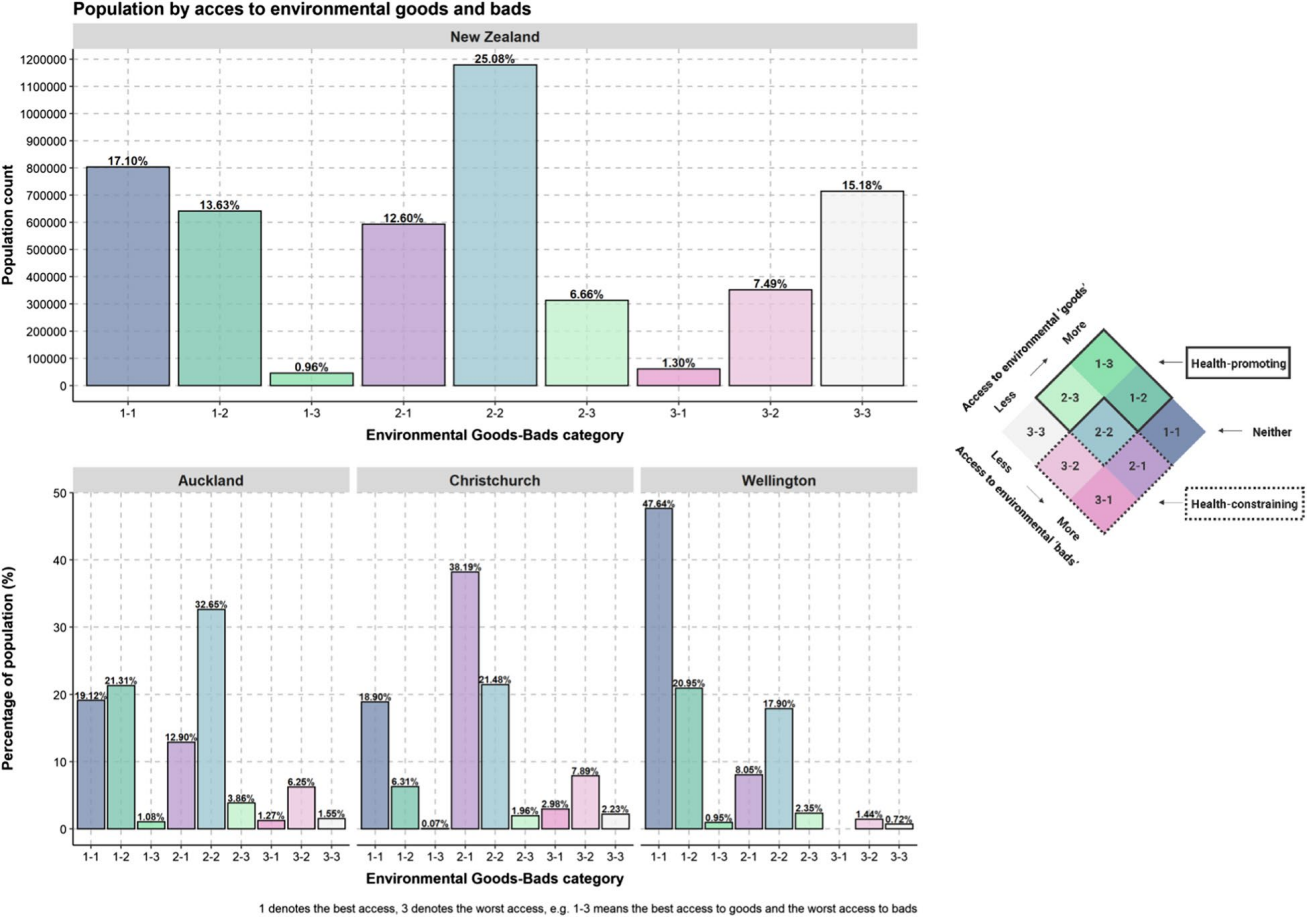
Healthy location index (HLI) category as a percentage of the population

Figure 6 shows the proportion of the New Zealand population residing within each HLI category based on the 2018 New Zealand census population count based on meshblock. Very few live at the extremes of the HLI; for instance, 0.96% (45,000 people) in 1–3 (most ‘goods’, least ‘bads’) and 1.30% (61,000 people) in 3–1 (least ‘goods’, most ‘bads’). In addition, population distribution by HLI also varies in three main urban centres. There is the highest proportion of the population living in balanced environments, having both ‘goods’ and ‘bads’ easily accessible, and one-quarter of the population living in health-promoting areas in Wellington. The pattern is less favourable in Christchurch, where half of the population lives in the health-constraining environment, and only 9% live in health-promoting conditions.

**Fig. 7:**
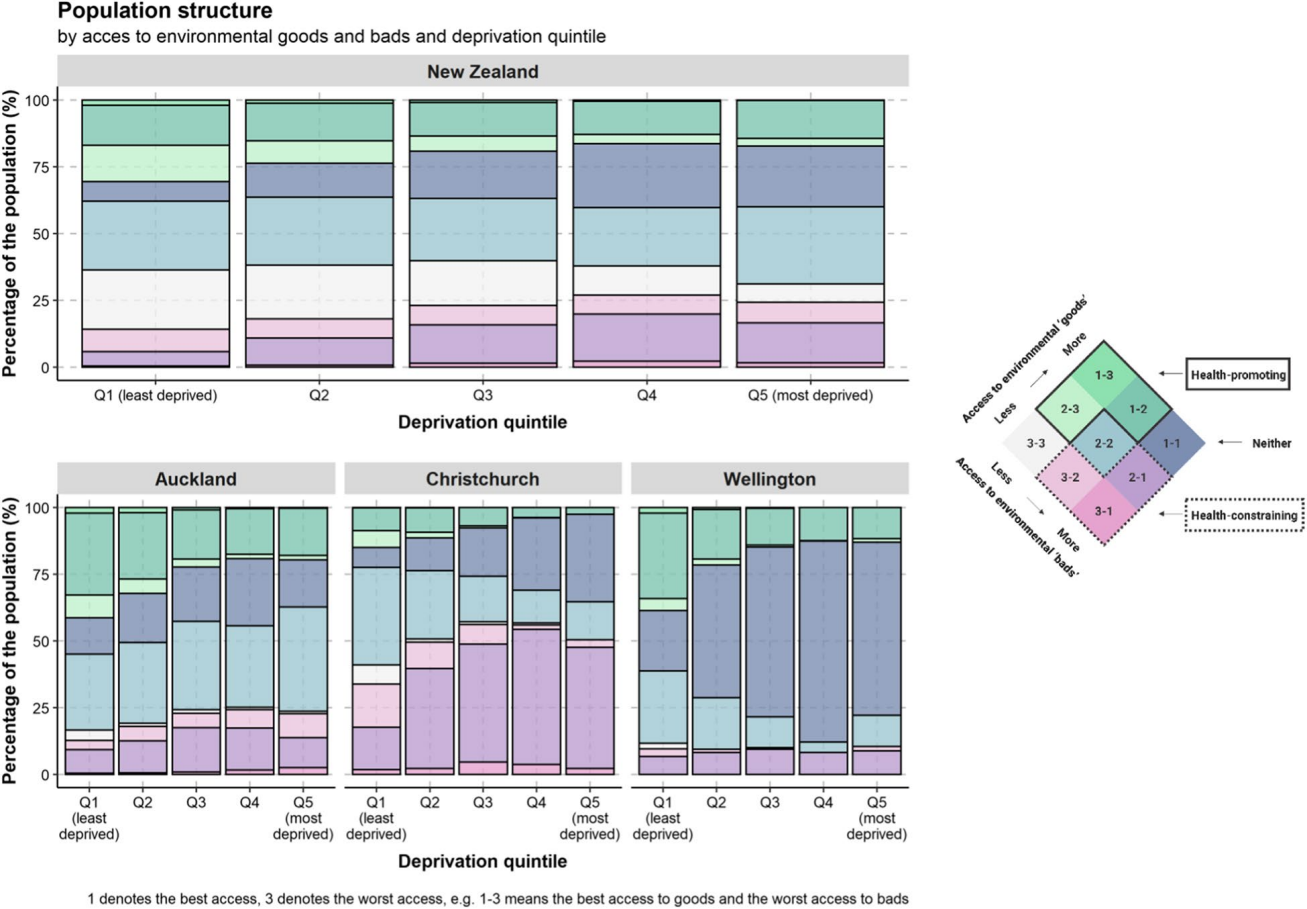
Healthy location index (HLI) category by area-level deprivation as a percentage of the population

Figure 7 shows the proportion of the population residing within each HLI category by area-level deprivation quintile. Overall, there was a social gradient in the HLI within New Zealand by area-level deprivation. For instance, there was a decrease overall in the proportion of environments classified as health-promoting (1–3, 2–3, and 1–2 categories) and an increase in the proportion of environments classified as health-constraining (2–1, 3–1 and 3–2 categories) as deprivation increased (Q1–Q5). There is an apparent social gradient of health-promoting environments in all three cities. Furthermore, throughout all deprivation categories, Wellington shows highly accessible ‘goods’ and ‘bads’, Auckland offers relatively balanced environments, and Christchurch shows a high proportion of people living in health-constraining environments (except Q1).

**Fig. 8:**
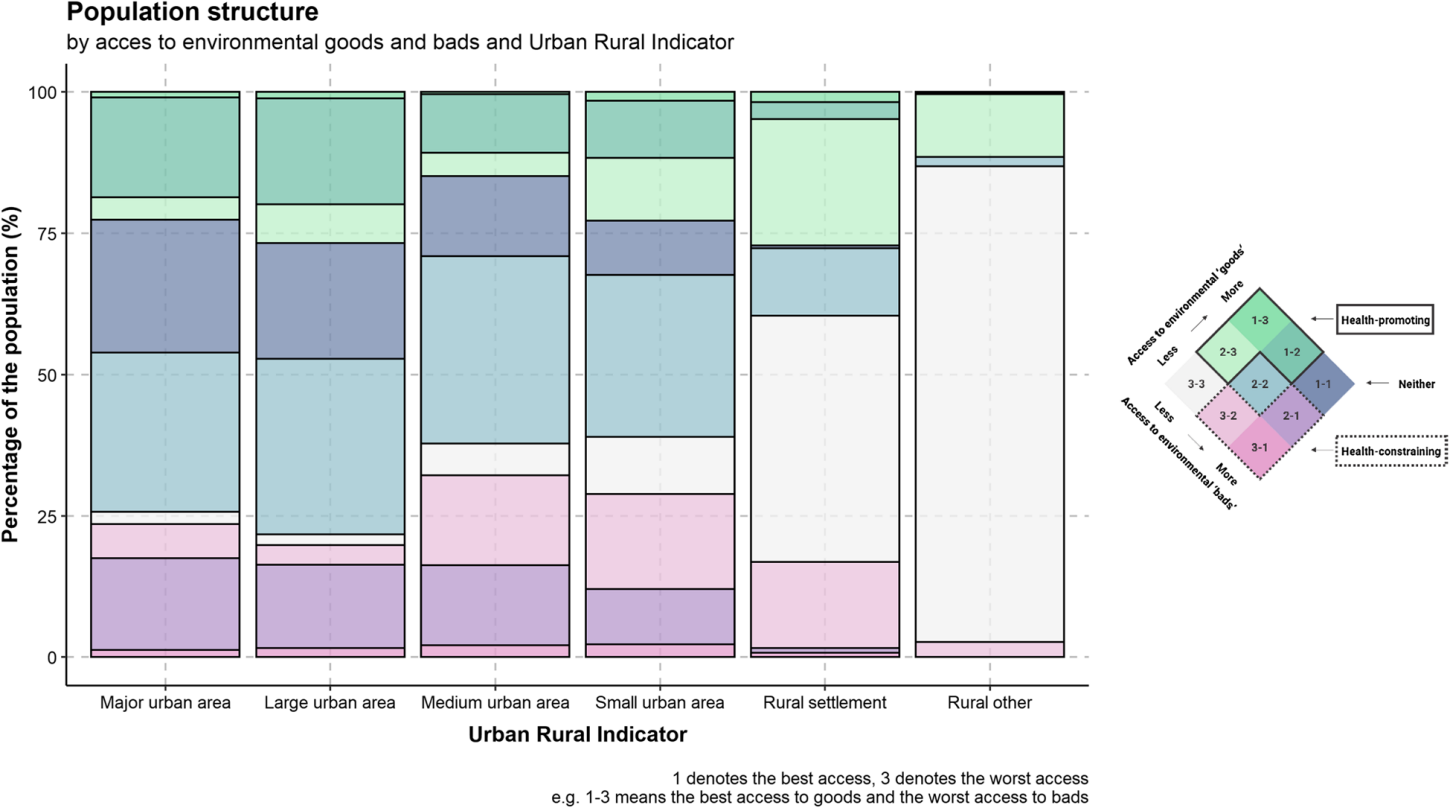
Healthy location index category by rural/urban classification as a percentage of the population

Figure 8 shows the proportion of areas by rural/urban classification by HLI category. As expected areas classified as major urban areas had a higher access to ‘goods’ and ‘bads’ and areas classified as rural had poorer access to both ‘goods’ and ‘bads’.In addition the proportion of population living in health-promoting environments is comparable between major and large urban and rural areas, while it is lower medium and small urban settlements.

## Discussion

This nationwide study firstly examined how health-promoting ‘goods’ and health-constraining ‘bads’ co-occur in space and secondly investigated socio-spatial patterning by area-level deprivation. We add to evidence by investigating a comprehensive measure of environmental ‘goods’ and ‘bads’ and produce novel healthy location index (HLI) comprising access to ten environmental features. Our findings show that both environmental ‘goods’ and ‘bads’ co-occur together and that features are patterned by area-level deprivation. Social gradients were displayed across environmental ‘goods ‘and ‘bads’. When considered in isolation, proximity to features largely decreased as area-level deprivation increased, with the exception of the most deprived decile, which had a slight increase in median distance. For instance, across all environmental ‘bads’, median proximity was almost double the distance in the least deprived, compared to the most deprived deciles. As such, we confirm and add to evidence that has suggested an overprovision of health-constraining ‘bads’ in deprived areas is a form of ‘environmental injustice’ [26, 70–72]. Even though such social gradients may contribute to inequity, our study shows that some deprived neighbourhoods may also have better access to environmental ‘goods’ such as physical activity facilities or supermarkets.

While much research considers ‘goods’ and ‘bads’ separately, we highlight the importance of considering the totality of environmental influences on behaviour and health, as only a few studies have previously [26, 52, 68, 73]. The HLI in this study suggests that the majority of deprived areas experience both an increase in environmental ‘bads’ and reduction in access to environmental ‘goods’. However, there are local variations of this pattern, for instance in most deprived areas of Wellington. In addition, considerably higher proportion of the population in small and medium urban areas live in predominantly health-constraining environments than in urban and rural settings. This finding coincides with research linking small urban areas of New Zealand with worsened health outcomes [74]. Results in this study further reinforce the need to embrace the multidimensional nature of neighbourhood and place when designing ‘healthy’ places alongside considerations of how these may interact with other factors such as deprivation or urbanicity of the area. Individual domains of the HLI also allow analysis of existing independent effects of environmental features on people’s health.

Our findings support previous evidence which not only highlights the need to focus on multiple facets of the environment [26, 52, 68], but also extend these considerations to show how this applies to both health-constraining and health-promoting environmental ‘bads’ and ‘goods’ at a nationwide level. Developing multidimensional measures of environmental influence will help capture the wider environmental influences on behaviour and health [68]. It is plausible that multiple environmental exposures act simultaneously and in isolation to influence geographical differences in health [48, 52]. Indeed, this effect has been highlighted previously by Macdonald [26]. For instance, an intervention to reduce smoking within Scotland by prohibiting tobacco use in pubs and bars, saw a reduction in alcohol consumption in moderate/heavy drinking smokers [75]. This is of importance because research has shown that the presence of a singular environmental ‘bad’ can put those individuals that reside in close proximity at greater risk of harm through adverse health behaviour [3, 5]. For instance, proximity to alcohol outlets alone are often enough to be associated with increased incidence of crime [3, 5, 76, 77] while increased availability of fast-food outlets has been associated with increased fast-food consumption and increased BMI [16, 78, 79], particularly in deprived populations [80–82]. Overall, related research utilising the index developed in this study [38] supports previous evidence [19, 26, 68, 73, 83] which shows how a simultaneous influence of multiple environmental ‘bads’ could extend to other facets of the environment including gaming venues, unhealthy food outlets, and alcohol outlets to adversely affect behaviour and health. Specifically, the HLI in this paper was related to both psychological distress and diagnosed mental health conditions [38].

We respond to previous calls to examine both health-constraining ‘bads’ and health-promoting ‘goods’ to show if areas with higher accessibility of tobacco, alcohol, gambling and fast food outlets are compensated by better access to environmental goods ‘ [26]. However, while we show closer proximity to a higher proportion of environmental ‘bads’ in deprived areas, other important aspects of neighbourhood that are not captured within this study (such as the social or quality of the environment) may exhibit important associations with behaviour and health [10, 30, 84, 85]. The quality of the environment in terms of aesthetics, safety, features, price, or choice for instance may be important in determining usage or behaviour. A recent UK study of parks highlighted the importance of this showing that despite more features and amenities such as play structures and benches existing with the more deprived areas there were also more safety concerns such as litter and graffiti present in parks in deprived areas [47].

The healthy location index shares similarities with previous research conducted in New Zealand and elsewhere. Several other studies [65, 67] utilised a similar approach to the topic evaluating both health-promoting and health-constraining environments at the same time. Sadler et al. [67] created raster-based analysis of the presence of environmental features combined with decision-making processes within one U.S. city. We build on and update the work of Pearson et al. [65] who utilised accessibility measures and similar data to create resilience index. However, our work not only uses more straightforward, extendable and replicable approach, but it also provides more spatial detail allowing for local analyses within cities and suburbs.

This paper relies on proximity to environmental ‘goods’ and ‘bads’ in the form of road and Euclidean distance to measure accessibility rather than other measures such as density. This is mainly due to the usage of small meshblock administrative units. Although meshblocks are comparable population-wise, calculated densities in most meshblocks would result in null values due to the high number of administrative units (n = 52,923). While the density of services can play an essential role in a highly urban environment, it is the simple presence or absence of a feature that is important in less urbanised settings. As mentioned earlier, New Zealand based research has shown that even access to a singular environmental ‘bad’ can put individuals in close proximity at greater risk of harm through adverse health behaviour [3, 5]. It is noteworthy that the distances to the closest individual domains are calculated from the population-weighted centroid of each meshblock in this study, not from individual dwellings.

The association between (built) environment and population health outcomes is not always clear and scientific evidence is often mixed [16, 30, 86]. While previous research has found that density and proximity metrics are largely comparable [87], this may be dependent on the environmental feature itself, how it is defined [88, 89], and the study context. While our index is based on proximity expressed by road network and Euclidean distance, similar to [73, 90, 91], other methodological approaches to measuring the environment include the use of relative measures, effectively comparing the number of healthy and unhealthy outlets to produce a ratio [59, 92], or utilise exposure as density-based measures [93, 94], or spatial access [95]. Even though the latter seems to provide a more complex representation of the real environment, measures often correlate with proximity [87], showing associations with health outcomes that are often location and context-specific [96, 97]. While relative and density-based measures have many benefits, they can mask detail and are often dominated by the quantity of retailers in urban environments [98], particularly unhealthy retailers. It is also conceptually challenging when trying to model environmental features that cover a range of vector types (e.g. points and polygons). Interestingly, research exploring the use of absolute and relative measures of the environment by comparing the use of simple exposure metrics (e.g. density) and those that are more complex (e.g. ratios of spatial access) have noted that more complex measures do not produce stronger associations with health behaviours [95]. Both approaches have valid scientific reasoning, however they still do not account for the actual utilisation of services where diversity of services [99] or habits [100] may be important factors in the decision-making processes.

Therefore, instead of using more complex methods such as multivariate statistics, weighting or regression analysis [64, 66] to create the index, we have opted for a method of creating typology based on the combination of ranked domains. On the one hand, this approach provided a non-parametrical basis for the analysis as the accessibility of individual environmental features differs in the distribution. It also provided an easy to understand metric, allowing immediate comparison between domains. On the other hand, this method results in possible loss of information that served as a basis for the index, especially when combined with subsequent aggregation to deciles. Being aware of this, we made publicly available not only the values of HLI but also the distances and ranks that lead to the final index.

For the construction of HLI, we used ten domains from a seemingly infinite set of environmental features. We have selected these based on previous evidence [14, 30, 65, 101], long-term collaboration with policy-makers as well as the accessibility of the data in enough detail for the whole of New Zealand. Other domains that could be considered part of the index include walkability, land use and street typology, air pollution, noise, thermal capacity/comfort of area, precipitation, and many more environmental and socio-cultural features. Even though these were not included now, they can be easily added if suitable data are available in future. Another limitation of our index is its binary view of the nature of domains describing them as either ‘goods’ or ‘bads’. While some of the selected domains are undoubtedly health-constraining environmental ‘bads’ (e.g. alcohol outlets, gaming venues) or health-promoting environmental ‘goods’ (fruit and vegetable outlets, blue space), the classification or perception of others might be mixed or local specific. For instance, green space is predominantly considered health-promoting [31, 34, 35], but some studies have shown mixed findings between green space and health outcomes [34, 36] or even found negative relationships for certain types of green space [32, 102].

Ideally, the research on the built and food environment should be conducted in a systemic way [103], considering that food outlets can also vary greatly with regard to their definition, range of food options and relationship with health outcomes. Although a predominant feature of the existing literature, this makes a binary classification of food outlets limiting as the nature of food provision is multifaceted [103] and there are increasing attempts to provide healthy options even within the fast food industry. However, in the New Zealand environment, fast food outlets are considered mostly health-constraining as evidenced by national [104] and international [105] classifications and policies, and a nationwide survey of food environments and policies that shows the majority of food and beverages promoted and sold are still of an unhealthy nature [106, 107]. In addition, the serving size and energy density of New Zealand fast food products has increased significantly [108]. While some fast food and takeaway outlets may provide healthy food options or can solely focus on healthy food; the choice is up to individual behaviour of the customer that we were not able to capture in the development of the index as such data are not available. Aligning with previous research [13–16] we categorised supermarkets as environmental ‘goods’ as they provide access to fresh produce such as fruit and vegetables, even though they also sell alcohol and tobacco. An example of local specific differences are dairies/convenience stores, in New Zealand settings, we classified them as *‘*bads’ due to their focus on selling ready-made energy-dense food products. However, their focus may be different in other parts of the world.

This paper aims to comprehensively describe the development of HLI and it provides socioeconomic and demographic summaries of the index however, it does not include any health outcomes testing the usability of the index in health-related context. However, a recent study [38] that used the HLI showed strong associations between geographic access to health-constraining environmental ‘bads’ and a lack of access to health-promoting environmental ‘goods’ and diagnosed mental health conditions and self-reported psychological distress. Mirroring findings from UK evidence [68] those environments with most ‘bads’ and least ‘goods’ were related to adverse outcomes for both diagnosed mental health and psychological distress [38]. Nevertheless, future research should test the use of this index alongside other health outcomes and behaviours in New Zealand. That is why, all data including details on individual domains (distances to the closest feature, ranks, and deciles) and HLI are openly available at https://www.canterbury.ac.nz/science/research/geohealth/publications-reports-and-data/ under Data/Environmental Goods and Bads [109]. While accessibility does not necessarily equate to utilisation, we do not have data on the latter and are therefore unable to model that level of complexity at this stage. Therefore, future research may wish to develop multiple measures of exposure, availability and access to ensure comparability between measures all of which have previously shown to be highly correlated [110, 111]. Moreover, the future research would benefit from exploring exposure (i.e. HLI), behaviour (i.e. physical activity), outcome associations (i.e. mental health) by exploring mediating pathways or mechanisms rather than assuming exposure is associated with outcome without the any explanatory pathways to define how such a relationship may operate.

## Conclusion

Due to the collaboration with the New Zealand Ministry of Health, our findings aim to help policymakers to develop appropriate strategies and interventions for those areas which are most deprived and which carry the greatest burden of disease. Interestingly, our findings show that while we observe greater accessibility to health-constraining environmental ‘bads’ in the more deprived areas we also see greater accessibility to some health-promoting environmental ‘goods’ such as physical activity facilities. However, overall, the HLI shows clear evidence that the most deprived areas of New Zealand often have the best access to environmental ‘bads’ and limited access to environmental ‘goods’. In addition, due to the HLI being available at meshblock level (the smallest geographic unit), the index is able to capture both inter-regional and local variations in accessibility to health-promoting and health-constraining environments and their combination. This research uses nationwide data on a comprehensive range of environmental features to show empirically for one of the first times at a nationwide level how access to environmental ‘goods’ could be utilised to offset the effects of environmental ‘bads’ particularly in deprived areas. Understanding this complexity and nuance is important in terms of public health interventions, and requires further research. The findings within this study highlight the multidimensional nature of place and help to begin to inform planning efforts for future modifications of the environment while considering the effects of deprivation as well as allowing for a better understanding of existing effects that the built environments have on people’s health. Data are openly available [109].

## Supplementary Information

**Additional file 1: **Geo-FERN (Geographic Information System Food Environment ReportiNg) Checklist. **Figure S1.** Boxplots (with jittered individual values) of distance (km) to health-constraining environmental ‘bads’ and health-promoting environmental ‘goods’ by decile of area-level deprivation. The y-axis has been transformed (pseudo-log) to cover full range of values.

## Data Availability

Data are available at https://www.canterbury.ac.nz/science/research/geohealth/publications-reports-and-data/.
